# Filopodia: A Rapid Structural Plasticity Substrate for Fast Learning

**DOI:** 10.3389/fnsyn.2017.00012

**Published:** 2017-06-20

**Authors:** Ahmet S. Ozcan

**Affiliations:** Machine Intelligence Laboratory, IBM Almaden Research CenterSan Jose, CA, United States

**Keywords:** filopodia, plasticity, fast learning, synaptogenesis, memory, spine growth, pruning, dendritic spines

## Abstract

Formation of new synapses between neurons is an essential mechanism for learning and encoding memories. The vast majority of excitatory synapses occur on dendritic spines, therefore, the growth dynamics of spines is strongly related to the plasticity timescales. Especially in the early stages of the developing brain, there is an abundant number of long, thin and motile protrusions (i.e., filopodia), which develop in timescales of seconds and minutes. Because of their unique morphology and motility, it has been suggested that filopodia can have a dual role in both spinogenesis and environmental sampling of potential axonal partners. I propose that filopodia can lower the threshold and reduce the time to form new dendritic spines and synapses, providing a substrate for fast learning. Based on this proposition, the functional role of filopodia during brain development is discussed in relation to learning and memory. Specifically, it is hypothesized that the postnatal brain starts with a single-stage memory system with filopodia playing a significant role in rapid structural plasticity along with the stability provided by the mushroom-shaped spines. Following the maturation of the hippocampus, this highly-plastic unitary system transitions to a two-stage memory system, which consists of a plastic temporary store and a long-term stable store. In alignment with these architectural changes, it is posited that after brain maturation, filopodia-based structural plasticity will be preserved in specific areas, which are involved in fast learning (e.g., hippocampus in relation to episodic memory). These propositions aim to introduce a unifying framework for a diversity of phenomena in the brain such as synaptogenesis, pruning and memory consolidation.

## Introduction

Learning and memory encoding in the brain are based on changes in synaptic connections between neurons. Current thinking on long-term memory has shifted from synaptic weight changes between previously existing neurons to rewiring neural networks through formation and elimination of synapses (Chklovskii et al., 2004; Holtmaat and Svoboda, 2009). Even though both processes can co-exist and are not mutually exclusive, their operation time-scales can be very different (Tetzlaff et al., 2012).

Neural rewiring offers a potentially massive capacity, compared to simple weight changes in a fixed network topology. However it comes at a cost of decreased speed in learning. Formation and elimination of functional synapses are inherently slow processes that can take hours to days (Knott et al., 2006; Le Bé and Markram, 2006), making them incompatible with fast learning (e.g., single-trial learning). For example, episodic memory (Henke, 2010), which refers to the autobiographical memories with temporal and spatial information, relies on rapid encoding of associations that can be “on the spot”. Compared to the slow, multiple-trial mode for skill development or semantic memory encoding, episodic memory requires rapid learning with one or few trials. Therefore, it is debatable whether fast learning is mainly based on the weight changes between existing synapses, a process in which the network topology is conserved.

One of the most thoroughly studied form of synaptic plasticity is long-term potentiation (LTP; Bliss and Collingridge, 1993; Malenka and Bear, 2004; Nicoll, 2017). In the broadest definition, LTP refers to a long-lasting enhancement in synaptic strength in response to brief, high-frequency stimulation. Long-term depression (LTD) is a complimentary process, which weakens the synaptic strength. The LTP process is typically divided into early and late phases. The early phase of LTP, which lasts about 60 min, is independent of protein synthesis and involves the persistent activation of protein kinases. The late phase of LTP, which may last many hours or days (even months have been reported), requires new gene transcription and mRNA translation. Even though LTP has been observed in different parts of the brain, most of the learning and experimental evidence are based on the studies of the hippocampus, which is essential for episodic memory (Eichenbaum, 2000; Zeidman and Maguire, 2016). In the hippocampal CA3 area, there is evidence of LTP induction within seconds of a single presentation of a pattern (Bliss and Collingridge, 1993), supporting the view that synaptic plasticity is the basis of rapid learning and memory encoding.

A recent *in vivo* study (Attardo et al., 2015) of hippocampal excitatory synaptic lifetimes reports surprising results. Namely, nearly 100% of the connections in the observed CA1 area of adult mice were replenished after few weeks, supporting the transient nature of hippocampal memory. On the other hand, about 60% of the synaptic connections in the neocortex were stable in the same study. These findings support the idea that structural plasticity is an underlying element of rapid learning and encoding of episodic memories. If LTP and LTD were the only fundamental neural correlates for rapid memory encoding, it would not be expected to see a massive restructuring of the hippocampal network as reported by Attardo et al. (2015). Even though other studies (Engert and Bonhoeffer, 1999; De Roo et al., 2008) also observed structural changes (i.e., growth of new protrusions and synapse formation) following LTP, some of these were related to the existing connections and resulted from perforated and branched spines (Geinisman, 1993; Toni et al., 2001). Furthermore, according to Watson et al. (2016), LTP in the adult hippocampus produces synapse enlargement and prevents the formation of new dendritic spines. These findings support the view that LTP is fundamentally a form of synaptic plasticity rather than a process that promotes the rewiring of the neural network. However, LTP induction was followed by the addition of new spines, in the young subjects (P15), suggesting a different effect on the network structure. These diverse results from different studies beg for a fresh look at plasticity in the context of brain development and memory. Especially focusing on fast learning (e.g., learning based on single exposure) could be the key to identify and distinguish the mechanisms, since in multi-trial, repetitive learning, different forms of plasticity can be encompassed due to the longer duration.

## Plasticity and Stability

Memories are formed by learning. Therefore, fast learning implies a rapid accumulation of new memories. During new memory encoding, existing memories must be protected from being overwritten, which brings up the plasticity-stability dilemma (Abraham and Robins, 2005; Mermillod et al., 2013). For optimal learning speed and memory retention, plasticity and stability need to be well balanced. This is a greater challenge for fixed topology neural network models, which only consider synaptic weight changes during learning. There has been theoretical attempts to address the trade-off between memory lifetime and synaptic strength (Fusi et al., 2005; Benna and Fusi, 2015; Kastner et al., 2016). Two-stage model of memory is a widely-supported solution for this challenge (McClelland et al., 1995; Frankland and Bontempi, 2005). This model separates memory into two systems that can compartmentalize stability and plasticity in different partitions (Roxin and Fusi, 2013). By employing periodic consolidation, new memories can be integrated into the stable system without overwriting the old memories. Hippocampus and neocortex are usually implicated for these roles, providing plasticity and stability, respectively. Furthermore, there is good evidence for memory consolidation during sleep (Stickgold, 2005; Diekelmann and Born, 2010).

In addition to multiple memory systems, introducing complexity into synaptic plasticity in the form of metaplasticity (Abraham, 2008) can also improve memory performance by allowing synapses to operate at multiple time-scales (Roxin and Fusi, 2013). Metaplasticity can be described as the “plasticity of synaptic plasticity” (Abraham, 2008). Even though it can entail an extensive range of mechanisms, the standard paradigm is the balance of synaptic weight changes involving the LTP and LTD processes. Given the growing experimental evidence for dendritic spine population dynamics, the scope of metaplasticity will undoubtedly grow and include structural plasticity mechanisms.

## Developmental Perspective for Learning and Memory

Human brain goes through a complex trajectory of development (Greenough et al., 1987) especially in the postnatal years. Since there are well known differences in the learning ability and the memory performance between young children and adults (Janacsek et al., 2012), one must also take into account the different phases of brain development and processes governing learning and memory encoding. The first few postnatal years is marked by excessive synapse formation and this period is usually referred to as “synaptogenesis” (Bianchi et al., 2013). After reaching a plateau in synaptic levels, pruning commences which directs the selective removal of synapses in the neural network up to 40%–50% of the peak density. Time course for synaptogenesis and pruning vary highly between species and even between different regions of the human brain (Bourgeois et al., 1994; Huttenlocher and Dabholkar, 1997). For example in the macaque brain, synaptogenesis peaks during infancy and pruning ends synchronously in all parts (Rakic et al., 1986). For humans, synaptogenesis is prolonged and pruning continues until late adolescence in most parts of the neocortex with significant delays in the prefrontal cortex (Bianchi et al., 2013).

**Figure 1:**
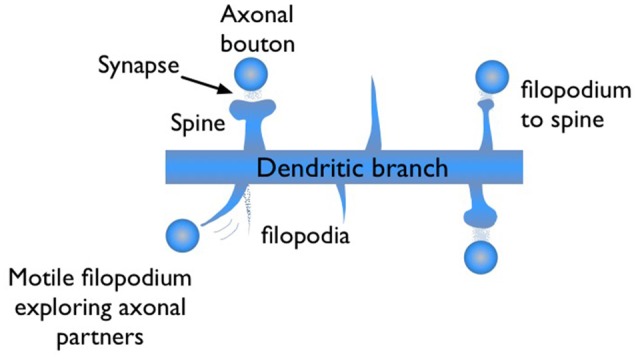
Illustration of filopodia and spine morphologies on a dendritic branch with axonal boutons nearby. Filopodia are motile long, needle like protrusions which grow and retract within minutes. When a filopodium makes a synaptic contact, its morphology may evolve from “thin spine” to “mushroom” shape, indicating maturation.

During synaptogenesis, dendritic branches of excitatory neurons show abundant numbers of needle-like spikes, called filopodia (Ziv and Smith, 1996; Jontes and Smith, 2000; Hering and Sheng, 2001; Matus, 2005; Zuo et al., 2005). These protrusions are highly motile structures with short lifetimes, on the order of minutes to hours. Some of these filopodia evolve into mushroom shape dendritic spines, which are the stable synaptic connection sites of dendrites (Figure 1). Dendritic spines are the postsynaptic sites for the vast majority of excitatory synapses in cortex. Therefore, generalizations based on spine dynamics can be made in regards to learning and memory.

Even though a large number of studies exist on dendritic spines, the development process of spines and the fundamental role of filopodia are still debated. In a review article, Yuste and Bonhoeffer (2004) discuss the potential functional role of filopodia in a section titled “Dendritic filopodia: structure looking for function”. They mention that the filopodia are potentially the precursors to spines during synaptogenesis but it is rare to find them on mature neurons. This finding suggests that their role is mainly developmental. For example, studies on mice report more than 50% of dendritic protrusions as filopodia in the first 2 weeks after birth and only 2%–10% in the adult barrel cortex (Portera-Cailliau et al., 2003; Zuo et al., 2005). In some cases, filopodia were “virtually absent (i.e.,<1%)” in the adult (Grutzendler et al., 2002).

Other experimental evidence shows that the role of filopodia in the brain is not exclusively developmental, raising the question about their functionality outside the realm of synaptogenesis. Due to their length and motility, filopodia were suggested to play an exploratory role in searching axonal partners (Ziv and Smith, 1996; Jontes and Smith, 2000; Yuste and Bonhoeffer, 2004). Perhaps in support for this type of functionality, one study on the adult prefrontal cortex suggests a link between filopodia and cognitive abilities. According to *in vitro* studies on rhesus monkeys by Dumitriu et al. (2010), a large majority of dendritic spines in the cortical layer III have the filopodia morphology (thin, long and motile spines rather than short and mushroom like). Moreover, aging reduces only these types of thin spines, which is also linked to the decline in cognitive abilities. Another study by Petrak et al. (2005) provides evidence for synaptogenesis in mature hippocampal dendrites via filopodia and proliferation of these “immature spines” when the synaptic transmission is blocked. In addition to these results, Toni et al. (2001) observed an abundant number of filopodia in the adult dentate gyrus (DG). Since adult neurogenesis is a common phenomenon in DG (Deng et al., 2010), the filopodia growth on the newborn cells are not surprising. Therefore, additional *in vivo* studies are needed to understand the role and density of filopodia in the mature hippocampus to make more general conclusions.

**Figure 2:**
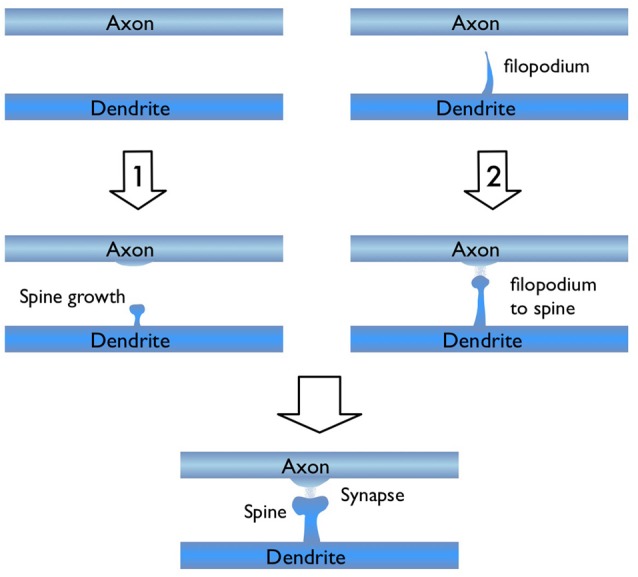
Illustration of two possible routes for excitatory synapse formation between an axon and a dendrite which belong to two parent neurons. Route 1 shows the activity dependent *de novo* spine growth. In route 2, a pre-existing filopodium evolves into a stable spine in response to neuronal activity.

**Figure 3:**
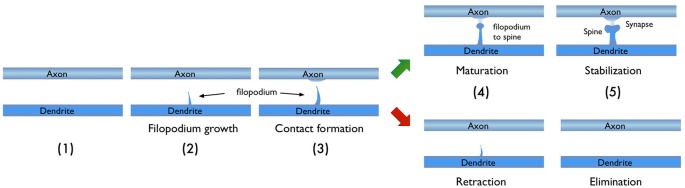
Typical stages in the growth of new dendritic spine. Depending on the persistence of the activity, the growth process may result in a new synaptic connection or failure to form one.

In light of the studies discussed above, I would like to focus on the process of new synapse formation and how filopodia can influence the plasticity rate. In Figure 2, two possible routes are illustrated, which lead to the formation of a stable spine and a synaptic contact between a dendritic branch and nearby axon. In the first route, there is no initial protrusion. A brand new dendritic spine has to grow *de novo* after the two parent neurons start “firing” in the Hebbian sense of learning. In the second route, there is an existing filopodium (which grew independent of the current activity) in close proximity, which transforms into a stable spine. Note that for both cases, the physical distance between the dendrite and the axon are assumed to be similar and less than the “touch” distance to form a synapse (i.e., they are appositions). If we define a hypothetical threshold to form a synaptic contact for a given apposition, then the filopodia route is clearly a lot closer to pass this threshold. For example, in a skill-learning or semantic memory task, one needs many trials over the course of the training time. Every trial helps to grow the spines and maintain the synaptic contact once it forms. If the number of trials is insufficient, the spine might retract or synapses might be eliminated (Figure 3). In this view, filopodia present a significant advantage in establishing a functional synaptic connection. Especially for single-trial learning (e.g., an episodic memory) being near the edge of the “threshold” would be very important.

## One Brain, Two Systems?

**Figure 4:**
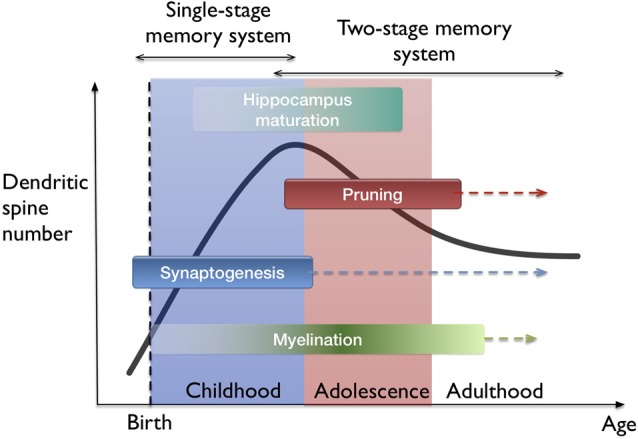
Lifetime trajectory of dendritic spine density and the developmental stages in the brain. Significant changes occur as synaptogenesis saturates which also coincides with the hippocampus maturation. Arrows indicate continuing processes throughout lifetime, such as synaptogenesis, but at much lower rates. On top, the hypothesis of single-stage to two-stage memory system transition is indicated.

The idea of filopodia providing a substrate for fast learning and structural plasticity prompts one to consider *where* and *when* in the brain does fast leaning occur. The developing neocortex in the early postnatal period as well as the hippocampus naturally fit in this picture. I hypothesize that both fast (single trial) and slow (many trial) learning in the developing brain are governed by a single-stage memory system, where filopodium-guided synaptogenesis (for excitatory synapses) provides a global and rapid mechanism of plasticity, supported by the rapid generation and elimination of filopodia. On the other hand, stability is conserved by the mature spines which operate at a different time-scale. In this view, slow learning and episodic memory functions start to get separated between the neocortex and the hippocampus. This interpretation supports the phenomenon of infantile amnesia (Travaglia et al., 2016) which refers to the inability of children in encoding or storing episodic memories before 2 years of age. The transition from a single-stage to two-stage memory system is probably gradual since the maturation of all hippocampal areas occur over several years (Lavenex and Banta Lavenex, 2013; see Figure 4). Also, the association-related cortical regions mature very late in humans (Miller et al., 2012). From the memory consolidation point of view, this can be described as a transition from a global “synaptic consolidation” to a “system consolidation”. Nevertheless, synaptic consolidation remains as a fast and local process within the system consolidation framework (Frankland and Bontempi, 2005).

The idea that filopodia and spines can play two different roles in rapid plasticity and stability is a type of metaplasticity argument. This is a metabolically expensive solution as it results in generating high numbers of new synapses and considering that most brain energy is used on synaptic transmission (Harris et al., 2012). Brain glucose uptake reaches adult levels by 2 years of age and peaks during childhood (Kuzawa et al., 2014). It peaks between the ages of 4–12 and decreases back to adult levels by the end of the second decade of life (Bentourkia et al., 1998). There is a similar trajectory for the development of spine density (Penzes et al., 2011). Therefore, there is a correlation between energy expenditure of the brain and spine density. In this regard, transition to a two-stage memory system with consolidation is probably a useful strategy in terms of brain’s energy consumption.

Another interesting comparison in regards to metabolic energy consumption is LTP based synaptic plasticity vs. filopodia driven plasticity. It is well known that LTP might be sustained for many hours, days and, in rare cases, even months (Abraham, 2003). LTP also causes an increase in glutamate response of a dendritic spine due to an increase in the number of α-amino-3-hydroxy-5-methyl-4-isoxazolepropionic acid (AMPA) receptors (Matsuzaki et al., 2004). Energetic calculations for neural computations (Howarth et al., 2012) show that a substantial portion of the cerebral energy expenditure (~50%) is used for neuronal signaling via the postsynaptic glutamate receptors, suggesting that LTP might be a metabolically expensive form of plasticity. For filopodia based connections, the caveat could be the functionality of the synapse after axonal contact. Filopodia contain little or no AMPA receptors (Matsuzaki et al., 2001). If most filopodia-initiated contacts are not functional (i.e., AMPA silent), it might not be prohibitive to form an abundant number of these thin protrusions from an energy expenditure standpoint. Furthermore, the majority of these are selectively pruned before they evolve into stable functional synapses. On the other hand, filopodia are rapidly growing, motile structures which require significant amount of actin dynamics. The actin cytoskeleton turnover is considered to be a part of the “housekeeping” (i.e., non-signaling) processes and there is an on-going debate about the contribution of actin dynamics to the overall energy expenditure. According to the recent findings of Engl et al. (2017), actin turnover can be a significant energy drain, contributing 25% to the rate of oxygen consumption in their measurements. This study was based on rat brain tissues obtained from subjects at the early developmental stage (P10) and, therefore, potentially reflect the energy expenditure of spine and filopodia motility.

We should clarify that the previous comment on the energy efficiency of a two-stage memory with periodic consolidation is not contradictory to the discussion on filopodia. A globally plastic brain with an overall trend of synaptogenesis is certainly a more energy demanding model. However, the highly protrusive activity of filopodia may not be as costly as it appears, since the majority (>90%) of filopodia do not survive and most of them lack AMPA receptors. Given the developing understanding of brain’s energy budget, more detailed models and measurements are needed to make more conclusive comments on this issue.

## Discussion

Considering cortical rewiring as the basis of learning, key questions arise related to the speed of plasticity and the stability of memories. The physiological processes underlying structural plasticity can be orders of magnitude slower than the mechanisms involved in synaptic plasticity. Therefore, the interplay of synaptic and structural plasticity has not been sufficiently tackled to provide a holistic theory on the physiology of learning and memory. Computational models with artificial neural networks in the era of “deep learning” (LeCun et al., 2015) exclusively focus on synaptic plasticity (i.e., weight adjustments). These models are based on older neural models that were developed in a period when there was no strong experimental evidence for cortical rewiring. Since synaptic potentiation occurs within seconds and can sustain for many minutes, LTP can be a sufficient mechanism to explain how new memories can form rapidly (Bliss and Collingridge, 1993), even for “one-shot” learning and without changing the network structure. However, it is clear that structural plasticity is an essential mechanism not just reserved for the developing brain, or for the post-injury period, or for a specific memory paradigm (e.g., procedural memory). Therefore, it is necessary to combine these fragmentary concepts to understand the structural and molecular changes in the brain which underpin learning and memory.

Spine morphology and dynamics are key to understand structural plasticity, since dendritic spines are the basis for most synaptic connections. Rich morphological variety and the dynamic nature of spines make them difficult to study and the results from different experiments may even appear contradictory. For example, the classification of filopodia and spines are based on morphological appearance, which can be subjective. Besides, the age of the subjects as well as the cerebral location vary in each study, further complicating the task to make general assumptions for spine development mechanisms (see Table 1).

**Table 1:** Summary of time-dependent studies of filopodia mentioned in the text, ordered chronologically by publication date.

Reference	Subject	Method	Age	Location	Imaging	Filopodia dynamics
Dailey and Smith (1996)	rat	*in vitro*	2–7 days old	hippocampus (CA1–CA3)	time-lapse microscopy (0.5, 1 and 5 min intervals)	~2.5 μm/min extension rate (median lifetime 10 min)
Ziv and Smith (1996)	rat	*in vitro*	3–4 days old initially 9–11 days *in vitro* 20–27 days *in vitro*	hippocampus (CA1–CA3)	time-lapse microscopy (3 min resolution)	mean lifetime 9.5 min
Xu et al. (2009)	mouse	*in vivo*	1–5 month old	motor cortex	two-photon microscopy (data taken on days 1, 2, 4, 8, 16)	majority of filopodia turn-over within 1 day
Grutzendler et al. (2002)	mouse	*in vivo*	1 month old and adult (4–5 month)	visual cortex (layer 5)	two-photon microscopy (hourly measurements)	“‥majority extended or retracted during the 4 h period” ~100% turnover within 3 days
Roelandse et al. (2003)	mouse	*in vitro*	young (3 week old) and young adult (5 week old)	hippocampus (CA1)	time-lapse microscopy (10 s resolution)	growth and retraction within 1 min (videos show dramatic changes every 10 s)
Zuo et al. (2005)	mouse	*in vivo*	1 month old and adult (4–5 month)	cortex (barrel, motor, prefrontal)	two-photon microscopy (hourly measurements)	~20% of filopodia turnover within 1 h
Toni et al. (2001)	mouse	*in vitro*	adult	hippocampus (DG)	time-lapse microscopy (30 min intervals)	new growth and retraction of filopodia within 30 min
Lohmann and Bonhoeffer (2008)	mouse and rat	*in vitro*	postnatal 0–2 days	hippocampus (CA3)	time-lapse microscopy (3.9 Hz acquisition frequency)	filopodia growth (axon contact) <1 min (functional synapse establishment 30–120 min)

*Table highlights the diversity of subjects, age and location as well as the experimental techniques*.

Currently there are two main hypotheses that explain the function of filopodia (Jontes and Smith, 2000; Portera-Cailliau et al., 2003; Yuste and Bonhoeffer, 2004). The first hypothesis views filopodia as precursors to spines (i.e., a morphological stage in the spine growth process). The second hypothesis is the probabilistic function of filopodia, which postulates that the high number of long and motile protrusions (i.e., the filopodia) serve as “explorers” for axonal partners to increase the chance of synapse formation between neurons. The divergent roles for filopodia and spines were also suggested by Jontes and Smith (2000), who proposed that filopodia can facilitate the “burning-in” process (i.e., guiding synaptogenesis) whereas spines are mostly associated with existing connections. They also warn that the high levels of plasticity driven by the filopodia could be disruptive and chaotic. Therefore, the synaptic remodeling in the mature organism may approximate “weight adjustments”.

In this work, I hypothesize that dendritic filopodia lower the threshold and reduce the time to from new stable spines and synapses. This perspective is related to, but different than the existing postulates on filopodia functionality. The exploration of axonal partners via the growth of filopodia implies an advantage of random sampling in addition to activity driven synapse formation. The other functional role, spinogenesis, is related to the morphological evolution of a spine. Here, I also emphasize the link between filopodia-driven plasticity and the memory systems in a developmental framework. Existing studies have shown that dendritic branches employ abundant numbers of filopodia in the developing brain, which grow and retract within minutes (Dailey and Smith, 1996; Roelandse et al., 2003; Lohmann and Bonhoeffer, 2008). Therefore, they may provide a route for rapid memory encoding with one or few trials. As it has been suggested before, by increasing the capture cross-section of dendrites, filopodia also play an active role in exploring the network around them before choosing synaptic partners. Compared to growing new spines from dendritic shafts and testing new connections, this is a faster and more efficient strategy from a metabolic standpoint. In this paradigm, the main difference between a filopodia-based and an LTP-based plasticity is the lack of long-term commitment. During LTP, a specific axon-dendrite connection displays persistent activity that can last for many hours or days. This is a long-term commitment for the pre- and post-synaptic partners and may constitute the basis of memory storage (e.g., a transient memory in the hippocampus) in the form of synaptic efficacy. In the developing brain, when the neural circuits are not mature, exploration without long-term commitment may represent a better approach. It should also be emphasized that the filopodia-based plasticity is not necessarily an alternative to the LTP process. During the morphological evolution from filopodia to mature spines, LTP might play a major role to develop functional synaptic contacts. For example *in vitro* studies by Lohmann and Bonhoeffer (2008) reported that filopodia growth and axonal contact can occur within a minute. However, functional synapse establishment at those sites typically takes 30–120 min. In this context, LTP is not driving new spine growth but it occurs in response to it.

Although filopodia are abundant on the dendrites in the developing brain, they are rarely reported in mature brain studies. However, as previously pointed out, there are examples of filopodia-rich dendrites in the adult prefrontal cortex and hippocampus. Therefore it can be viewed as an available tool for neurons to regulate spine development rapidly. A good example of this regulation is hinted by the estrogen studies in the prefrontal cortex and in the hippocampus (Srivastava, 2012; Phan et al., 2015), which report dramatic increase in spine densities (i.e., filopodia suggested by their morphologic description) within minutes in response to estrogen exposure.

As seen in Table 1, sampling intervals that exceed the time resolution required to monitor changes happening within seconds is a limitation of the experimental framework of existing studies. The other limitation of the existing literature is the differences between *in vitro* and *in vivo* studies, which may include additional factors that may affect the spine and filopodia dynamics. Knott et al. (2006) comments on these differences, where their *in vivo* results showed significantly prolonged (i.e., >1 day) synapse formation, compared to the *in vitro* results that report synapse formation within 1–2 h after axo-dendritic contact.

Reviewing the spine and filopodia dynamics throughout the brain development, there are interesting correlations that indicate an efficient strategy for learning and memory processing. I hypothesize that the brain has a single-stage, globally plastic memory system until the hippocampus fully develops. The balance between fast plasticity and stability is enabled by multiple time-scales for synapse formation, especially facilitated through the filopodia-driven plasticity and the stability of the mushroom-shaped spines. Once the hippocampus becomes mature, the brain moves to a two-stage memory system with periodic consolidation. I further posit that filopodia still play a role for fast learning in the mature brain, but only in regions where this could be beneficial, such as in the hippocampus and in the prefrontal cortex. This role is complementary to other synaptic plasticity processes such as LTP and LTD.

The ideas presented here clearly need to be tested with *in vivo* studies where filopodia and spine dynamics are monitored carefully at short and long time scales. In the framework I hypothesize, controlling the filopodia density can have therapeutic implications in neuropsychiatric disorders where abnormal spine densities are observed (e.g., schizophrenia). Hence, understanding and modeling the functionality of filopodia can open up new clinical possibilities as well.

I would like to conclude by summarizing the following testable hypotheses presented in this article:

- Regulation of filopodia density in the developing brain (by genetic or pharmacological means) could affect learning and memory encoding performance, especially for tasks that depend on single exposure. A potential genetic approach to regulate the filopodia density may involve copine-6, which is a novel modulator of dendritic spine morphology. Studies by Burk et al. (2017) show that overexpression of copine-6 increases mushroom spines and dramatically decreases the filopodia density. In contrast, copine-6 knockdown has the opposite effect and significantly increases the number of filopodia. Alternatively, pharmacological means (e.g., latrunculin) can target actin dynamics to alter filopodia density.
- Long term trajectory of spine density can be influenced by interventions in filopodia density. This is based on the concept of filopodia-based plasticity in the developmental context. The interventions to alter filopodia density may include the use of the above mentioned genetic or pharmacological approaches.
- Differential roles of filopodia (rapid structural) and LTP (rapid synaptic) plasticity can be investigated by *in vivo* monitoring of different synaptic circuits. For example, the CA3-CA1 synapses (Schaffer collaterals) vs. EC-CA1 synapses in the hippocampus may provide fertile ground to examine rapid structural and synaptic plasticity mechanisms simultaneously.

## Author Contributions

ASO conceived the ideas in this work and wrote the manuscript.

## Conflict of Interest Statement

The author declares that the research was conducted in the absence of any commercial or financial relationships that could be construed as a potential conflict of interest. The author is an employee of IBM Research. This study was fully funded by IBM Research.

## References

[B1] Abraham W. C. (2003). How long will long-term potentiation last? Philos. Trans. R. Soc. Lond. B Biol. Sci. 358, 735–744. 10.1098/rstb.2002.1222 12740120 PMC1693170

[B2] Abraham W. C. (2008). Metaplasticity: tuning synapses and networks for plasticity. Nat. Rev. Neurosci. 9:387. 10.1038/nrn2356 18401345

[B3] Abraham W. C. Robins A. (2005). Memory retention—the synaptic stability versus plasticity dilemma. Trends Neurosci. 28, 73–78. 10.1016/j.tins.2004.12.003 15667929

[B4] Attardo A. Fitzgerald J. E. Schnitzer M. J. (2015). Impermanence of dendritic spines in live adult CA1 hippocampus. Nature 523, 592–596. 10.1038/nature14467 26098371 PMC4648621

[B5] Benna M. K. Fusi S. (2015). Complex synapses as efficient memory systems. BMC Neurosci. 16:F1 10.1186/1471-2202-16-S1-F1

[B6] Bentourkia M. Michel C. Ferriere G. Bol A. Coppens A. Sibomana M.. (1998). Evolution of brain glucose metabolism with age in epileptic infants, children and adolescents. Brain Dev. 20, 524–529. 10.1016/s0387-7604(98)00040-0 9840673

[B7] Bianchi S. Stimpson C. D. Duka T. Larsen M. D. Janssen W. G. M. Collins Z.. (2013). Synaptogenesis and development of pyramidal neuron dendritic morphology in the chimpanzee neocortex resembles humans. Proc. Natl. Acad. Sci. U S A 110, 10395–10401. 10.1073/pnas.1301224110 23754422 PMC3690614

[B8] Bliss T. V. P. Collingridge G. L. (1993). A synaptic model of memory: long-term potentiation in the hippocampus. Nature 361, 31–39. 10.1038/361031a0 8421494

[B9] Bourgeois J.-P. Goldman-Rakic P. S. Rakic P. (1994). Synaptogenesis in the prefrontal cortex of rhesus monkeys. Cereb. Cortex 4, 78–96. 10.1093/cercor/4.1.78 8180493

[B10] Burk K. Ramachandran B. Ahmed S. Hurtado-Zavala J. I. Awasthi A. Benito E.. (2017). Regulation of dendritic spine morphology in hippocampal neurons by Copine-6. Cereb. Cortex [Epub ahead of print]. 10.1093/cercor/bhx009 28158493

[B11] Chklovskii D. B. Mel B. W. Svoboda K. (2004). Cortical rewiring and information storage. Nature 431, 782–788. 10.1038/nature03012 15483599

[B500] Dailey M. E. Smith S. J. (1996). The dynamics of dendritic structure in developing hippocampal slices. J. Neurosci. 16, 2983–2994. Available online at: http://www.jneurosci.org/content/16/9/2983.short 8622128 10.1523/JNEUROSCI.16-09-02983.1996 PMC6579052

[B13] Deng W. Aimone J. B. Gage F. H. (2010). New neurons and new memories: how does adult hippocampal neurogenesis affect learning and memory? Nat. Rev. Neurosci. 11, 339–350. 10.1038/nrn2822 20354534 PMC2886712

[B12] De Roo M. Klauser P. Garcia P. M. Poglia L. Muller D. (2008). Spine dynamics and synapse remodeling during LTP and memory processes. Prog. Brain Res. 169, 199–207. 10.1016/s0079-6123(07)00011-8 18394475

[B14] Diekelmann S. Born J. (2010). The memory function of sleep. Nat. Rev. Neurosci. 11, 114–126. 10.1038/nrn2762 20046194

[B15] Dumitriu D. Hao J. Hara Y. Kaufmann J. Janssen W. G. M. Lou W.. (2010). Selective changes in thin spine density and morphology in monkey prefrontal cortex correlate with aging-related cognitive impairment. J. Neurosci. 30, 7507–7515. 10.1523/JNEUROSCI.6410-09.2010 20519525 PMC2892969

[B16] Eichenbaum H. (2000). A cortical-hippocampal system for declarative memory. Nat. Rev. Neurosci. 1, 41–50. 10.1038/35036213 11252767

[B17] Engert F. Bonhoeffer T. (1999). Dendritic spine changes associated with hippocampal long-term synaptic plasticity. Nature 399, 66–70. 10.1038/19978 10331391

[B18] Engl E. Jolivet R. Hall C. N. Attwell D. (2017). Non-signalling energy use in the developing rat brain. J. Cereb. Blood Flow Metab. 37, 951–966. 10.1177/0271678X16648710 27170699 PMC5322833

[B19] Frankland P. W. Bontempi B. (2005). The organization of recent and remote memories. Nat. Rev. Neurosci. 6, 119–130. 10.1038/nrn1607 15685217

[B20] Fusi S. Drew P. J. Abbott L. F. (2005). Cascade models of synaptically stored memories. Neuron 45, 599–611. 10.1016/j.neuron.2005.02.001 15721245

[B21] Geinisman Y. (1993). Perforated axospinous synapses with multiple, completely partitioned transmission zones: probable structural intermediates in synaptic plasticity. Hippocampus 3, 417–433. 10.1002/hipo.450030404 8269034

[B22] Greenough W. T. Black J. E. Wallace C. S. (1987). Experience and brain development. Child Dev. 58, 539–559. 10.2307/1130197 3038480

[B23] Grutzendler J. Kasthuri N. Gan W.-B. (2002). Long-term dendritic spine stability in the adult cortex. Nature 420, 812–816. 10.1038/nature01276 12490949

[B24] Harris J. J. Jolivet R. Attwell D. (2012). Synaptic energy use and supply. Neuron 75, 762–777. 10.1016/j.neuron.2012.08.019 22958818

[B25] Henke K. (2010). A model for memory systems based on processing modes rather than consciousness. Nat. Rev. Neurosci. 11, 523–532. 10.1038/nrn2850 20531422

[B26] Hering H. Sheng M. (2001). Dendritic spines: structure, dynamics and regulation. Nat. Rev. Neurosci. 2, 880–888. 10.1038/35104061 11733795

[B27] Holtmaat A. Svoboda K. (2009). Experience-dependent structural synaptic plasticity in the mammalian brain. Nat. Rev. Neurosci. 10, 647–658. 10.1038/nrn2699 19693029

[B28] Howarth C. Gleeson P. Attwell D. (2012). Updated energy budgets for neural computation in the neocortex and cerebellum. J. Cereb. Blood Flow Metab. 32, 1222–1232. 10.1038/jcbfm.2012.35 22434069 PMC3390818

[B30] Huttenlocher P. R. Dabholkar A. S. (1997). Regional differences in synaptogenesis in human cerebral cortex. J. Comp. Neurol. 387, 167–178. 10.1002/(SICI)1096-9861(19971020)387:2<167::aid-cne1>3.0.CO;2-Z 9336221

[B31] Janacsek K. Fiser J. Nemeth D. (2012). The best time to acquire new skills: age-related differences in implicit sequence learning across the human lifespan. Dev. Sci. 15, 496–505. 10.1111/j.1467-7687.2012.01150.x 22709399 PMC3383816

[B32] Jontes J. D. Smith S. J. (2000). Filopodia, spines, and the generation of synaptic diversity. Neuron 27, 11–14. 10.1016/s0896-6273(00)00003-9 10939326

[B33] Kastner D. B. Schwalger T. Ziegler L. Gerstner W. (2016). A model of synaptic reconsolidation. Front. Neurosci. 10:206. 10.3389/fnins.2016.00206 27242410 PMC4870270

[B34] Knott G. W. Holtmaat A. Wilbrecht L. Welker E. Svoboda K. (2006). Spine growth precedes synapse formation in the adult neocortex *in vivo*. Nat. Neurosci. 9, 1117–1124. 10.1038/nn1747 16892056

[B35] Kuzawa C. W. Chugani H. T. Grossman L. I. Lipovich L. Muzik O. Hof P. R.. (2014). Metabolic costs and evolutionary implications of human brain development. Proc. Natl. Acad. Sci. U S A 111, 13010–13015. 10.1073/pnas.1323099111 25157149 PMC4246958

[B36] Lavenex P. Banta Lavenex P. (2013). Building hippocampal circuits to learn and remember: insights into the development of human memory. Behav. Brain Res. 254, 8–21. 10.1016/j.bbr.2013.02.007 23428745

[B37] Le Bé J.-V. Markram H. (2006). Spontaneous and evoked synaptic rewiring in the neonatal neocortex. Proc. Natl. Acad. Sci. U S A 103, 13214–13219. 10.1073/pnas.0604691103 16924105 PMC1559779

[B38] LeCun Y. Bengio Y. Hinton G. (2015). Deep learning. Nature 521, 436–444. 10.1038/nature14539 26017442

[B39] Lohmann C. Bonhoeffer T. (2008). A role for local calcium signaling in rapid synaptic partner selection by dendritic filopodia. Neuron 59, 253–260. 10.1016/j.neuron.2008.05.025 18667153

[B40] Malenka R. C. Bear M. F. (2004). LTP and LTD: review an embarrassment of riches. Neuron 44, 5–21. 10.1016/j.neuron.2004.09.012 15450156

[B41] Matsuzaki M. Ellis-Davies G. C. R. Nemoto T. Miyashita Y. Iino M. Kasai H. (2001). Dendritic spine geometry is critical for AMPA receptor expression in hippocampal CA1 pyramidal neurons. Nat. Neurosci. 4, 1086–1092. 10.1038/nn736 11687814 PMC4229049

[B42] Matsuzaki M. Honkura N. Ellis-Davies G. C. R. Kasai H. (2004). Structural basis of long-term potentiation in single dendritic spines. Nature 429, 761–766. 10.1038/nature02617 15190253 PMC4158816

[B43] Matus A. (2005). Growth of dendritic spines: a continuing story. Curr. Opin. Neurobiol. 15, 67–72. 10.1016/j.conb.2005.01.015 15721746

[B44] McClelland J. L. McNaughton B. L. O’Reilly R. C. (1995). Why there are complementary learning systems in the hippocampus and neocortex: insights from the successes and failures of connectionist models of learning and memory. Psychol. Rev. 102, 419–457. 10.1037/0033-295x.102.3.419 7624455

[B45] Mermillod M. Bugaiska A. Bonin P. (2013). The stability-plasticity dilemma: investigating the continuum from catastrophic forgetting to age-limited learning effects. Front. Psychol. 4:504. 10.3389/fpsyg.2013.00504 23935590 PMC3732997

[B46] Miller D. J. Duka T. Stimpson C. D. Schapiro S. J. Baze W. B. McArthur M. J.. (2012). Prolonged myelination in human neocortical evolution. Proc. Natl. Acad. Sci. U S A 109, 16480–16485. 10.1073/pnas.1117943109 23012402 PMC3478650

[B47] Nicoll R. A. (2017). A brief history of long-term potentiation. Neuron 93, 281–290. 10.1016/j.neuron.2016.12.015 28103477

[B48] Penzes P. Cahill M. E. Jones K. A. VanLeeuwen J.-E. Woolfrey K. M. (2011). Dendritic spine pathology in neuropsychiatric disorders. Nat. Neurosci. 14, 285–293. 10.1038/nn.2741 21346746 PMC3530413

[B49] Petrak L. J. Harris K. M. Kirov S. A. (2005). Synaptogenesis on mature hippocampal dendrites occurs via filopodia and immature spines during blocked synaptic transmission. J. Comp. Neurol. 484, 183–190. 10.1002/cne.20468 15736233

[B50] Phan A. Suschkov S. Molinaro L. Reynolds K. Lymer J. M. Bailey C. D. C.. (2015). Rapid increases in immature synapses parallel estrogen-induced hippocampal learning enhancements. Proc. Natl. Acad. Sci. U S A 112, 16018–16023. 10.1073/pnas.1522150112 26655342 PMC4703013

[B51] Portera-Cailliau C. Pan D. T. Yuste R. (2003). Activity-regulated dynamic behavior of early dendritic protrusions: evidence for different types of dendritic filopodia. J. Neurosci. 23, 7129–7142. 12904473 10.1523/JNEUROSCI.23-18-07129.2003 PMC6740658

[B52] Rakic P. Bourgeois J.-P. Eckenhoff M. F. Zecevic N. Goldman-Rakic P. S. (1986). Concurrent overproduction of synapses in diverse regions of the primate cerebral cortex. Science 232, 232–235. 10.1126/science.3952506 3952506

[B53] Roelandse M. Welman A. Wagner U. Hagmann J. Matus A. (2003). Focal motility determines the geometry of dendritic spines. Neuroscience 121, 39–49. 10.1016/s0306-4522(03)00405-6 12946698

[B54] Roxin A. Fusi S. (2013). Efficient partitioning of memory systems and its importance for memory consolidation. PLoS Comput. Biol. 9:e1003146. 10.1371/journal.pcbi.1003146 23935470 PMC3723499

[B55] Srivastava D. P. (2012). Two-step wiring plasticity—a mechanism for estrogen-induced rewiring of cortical circuits. J. Steroid Biochem. Mol. Biol. 131, 17–23. 10.1016/j.jsbmb.2012.01.006 22349412

[B56] Stickgold R. (2005). Sleep-dependent memory consolidation. Nature 437, 1272–1278. 10.1038/nature04286 16251952

[B57] Tetzlaff C. Kolodziejski C. Markelic I. Wörgötter F. (2012). Time scales of memory, learning and plasticity. Biol. Cybern. 106, 715–726. 10.1007/s00422-012-0529-z 23160712

[B58] Toni N. Buchs P. A. Nikonenko I. Povilaitite P. Parisi L. Muller D. (2001). Remodeling of synaptic membranes after induction of long-term potentiation. J. Neurosci. 21, 6245–6251. 11487647 10.1523/JNEUROSCI.21-16-06245.2001 PMC6763190

[B59] Travaglia A. Bisaz R. Sweet E. S. Blitzer R. D. Alberini C. M. (2016). Infantile amnesia reflects a developmental critical period for hippocampal learning. Nat. Neurosci. 19, 1225–1233. 10.1038/nn.4348 27428652 PMC5003643

[B60] Watson D. J. Ostroff L. Cao G. Parker P. H. Smith H. Harris K. M. (2016). LTP enhances synaptogenesis in the developing hippocampus. Hippocampus 26, 560–576. 10.1002/hipo.22536 26418237 PMC4811749

[B61] Xu T. Yu X. Perlik A. J. Tobin W. F. Zweig J. A. Tennant K.. (2009). Rapid formation and selective stabilization of synapses for enduring motor memories. Nature 462, 915–919. 10.1038/nature08389 19946267 PMC2844762

[B62] Yuste R. Bonhoeffer T. (2004). Genesis of dendritic spines: insights from ultrastructural and imaging studies. Nat. Rev. Neurosci. 5, 24–34. 10.1038/nrn1300 14708001

[B63] Zeidman P. Maguire E. A. (2016). Anterior hippocampus: the anatomy of perception, imagination and episodic memory. Nat. Rev. Neurosci. 17, 173–182. 10.1038/nrn.2015.24 26865022 PMC5358751

[B64] Ziv N. E. Smith S. J. (1996). Evidence for a role of dendritic filopodia in synaptogenesis and spine formation. Neuron 17, 91–102. 10.1016/s0896-6273(00)80283-4 8755481

[B65] Zuo Y. Lin A. Chang P. Gan W.-B. (2005). Development of long-term dendritic spine stability in diverse regions of cerebral cortex. Neuron 46, 181–189. 10.1016/j.neuron.2005.04.001 15848798

